# Anthropometric estimators of abdominal fat volume in adults with overweight and obesity

**DOI:** 10.1038/s41366-023-01264-x

**Published:** 2023-02-07

**Authors:** Sophia Michel, Nicolas Linder, Anna Linder, Tobias Eggebrecht, Alexander Schaudinn, Matthias Blüher, Arne Dietrich, Timm Denecke, Harald Busse

**Affiliations:** 1 Integrated Research and Treatment Center (IFB) Adiposity Diseases, Leipzig University Medical Center, Leipzig, Germany; 2 Department of Internal Medicine, Neurology and Dermatology, Division of Dermatology, Venerology and Allergology, Leipzig University Hospital, Leipzig, Germany; 3 Department of Diagnostic and Interventional Radiology, Leipzig University Hospital, Leipzig, Germany; 4 Helmholtz Institute for Metabolic, Obesity and Vascular Research (HI-MAG) of the Helmholtz Zentrum München at the University of Leipzig and University Hospital Leipzig, Leipzig, Germany; 5 Department of Internal Medicine, Neurology and Dermatology, Division of Endocrinology and Nephrology, Leipzig University Hospital, Leipzig, Germany; 6 Department of Visceral, Transplantation, Thoracic and Vascular Surgery, Division of Bariatric Surgery, Leipzig University Hospital, Leipzig, Germany

**Keywords:** Obesity, Obesity

## Abstract

**Background/Objectives:**

To evaluate anthropometric measures for the prediction of whole-abdominal adipose tissue volumes *V*_XAT_ (subcutaneous *V*_SAT_, visceral *V*_VAT_ and total *V*_TAT_) in patients with obesity.

**Subjects/Methods:**

A total of 181 patients (108 women) with overweight or obesity were analyzed retrospectively. MRI data (1.5 T) were available from independent clinical trials at a single institution (Integrated Research and Treatment Center of Obesity, University of Leipzig). A custom-made software was used for automated tissue segmentation. Anthropometric parameters (*AP*) were circumferences of the waist (WC) and hip (HC), waist-to-hip ratio (WHR), waist-to-height ratio (WHtR) and the (hypothetical) hip-to-height ratio (HHtR). Agreement was evaluated by standard deviations *s*_d%_ of percent differences between estimated volumes (using results of linear *AP*–*V*_XAT_ regression) and measured ones as well as Pearson’s correlation coefficient *r*.

**Results:**

For SAT volume estimation, the smallest *s*_d%_ for all patients was seen for HC (25.1%) closely followed by HHtR (25.2%). Sex-specific results for females (17.5% for BMI and 17.2% for HC) and males (20.7% for WC) agreed better. VAT volumes could not be estimated reliably by any of the anthropometric measures considered here. TAT volumes in a mixed population could be best estimated by BMI closely followed by WC (roughly 17.5%). A sex-specific consideration reduced the deviations to around 16% for females (BMI and WC) and below 14% for males (WC).

**Conclusions:**

We suggest the use of sex-specific parameters–BMI or HC for females and WC for males–for the estimation of abdominal SAT and TAT volumes in patients with overweight or obesity.

## Introduction

Obesity is one of the major healthcare problems today [1]. In 2016, there were worldwide more than 1.9 billion adults aged 18 years and over with overweight and over 650 million with obesity [2]. Overweight and obesity are closely associated with an increased overall mortality and morbidity, often caused by metabolic or cardiovascular diseases. Efforts and overall costs of treatment constitute a relevant socioeconomic burden [3–5].

Compartments of abdominal adipose tissue contribute differently to metabolic homeostasis. Their size and composition are important risk factors in the pathogenesis of the relevant diseases [6]. Visceral adipose tissue (VAT) is a highly dyslipidemic and atherogenic fat depot due to its endocrine activity and is associated with higher cardiometabolic risks [7]. Accumulation of subcutaneous adipose tissue (SAT), in contrast, has been found to be associated with a lower metabolic mortality [8]. Metabolic complications like lipotoxicity and insulin resistance may arise when fat is stored in ectopic regions like the liver or skeletal muscle [9, 10].

Quantitative diagnostic tests of abdominal adipose tissue ranges from simple anthropometric measures, like BMI, WC or HC, to highly accurate but currently less practical methods like magnetic resonance imaging (MRI). In times of limited health resources, there is a clear need for simple and reliable markers to stratify metabolic or cardiovascular risks and to support the characterization of clinical outcomes in patients with overweight or obesity. The BMI is generally used to define underweight, normal weight, overweight and different grades of obesity [11–13]. The main drawback of all anthropometric parameters is their inability to differentiate between lean and fat mass as well as between SAT and VAT [12–20].

Studies have already sought to identify anthropometric parameters that correlate best with adipose tissue volume, predominantly in patients with normal weight or overweight [21–25]. For patients with overweight or obesity, clear-cut analyses of adipose tissue compartments are still lacking. The objective of this study was therefore to compare the suitability of a number of simple anthropometric measures for the prediction of abdominal SAT, VAT and total adipose tissue (TAT) volumes in patients with overweight or obesity.

## Materials/subjects and methods

### Subjects

This work is a single-institution, retrospective analysis of 181 patients (108 women, 73 men) with overweight or obesity who had undergone MRI during different clinical trials of Leipzig University’s Integrated Research and Treatment Center of Obesity (IFB *AdiposityDiseases*). Research was carried out with IRB approval by the Faculty of Medicine (references 283/11-ff, 284/10-ff, 363/10-ff and 363/11-ff) and patients’ informed consent. Study subjects were selected by age (at least 18 years old) and BMI (at least 25 kg/m^2^). Datasets were then reviewed for image quality and full coverage of the SAT compartment on all individual slices before inclusion (see below).

### MRI examination and data analysis

All patients had been examined in supine position in a single 1.5-T MRI system (Achieva XR, Philips Healthcare, Best, Netherlands) using the integrated whole-body coil for signal reception. The essential image series for fat quantification was a simple dual-echo gradient-echo pulse sequence with echo times matching opposed-phase and in-phase conditions using the following parameters: 50 transverse slices (two stacks covering the abdominopelvic region between diaphragm and pubic symphysis), slice thickness 10 mm thick, interslice gap 0.5 mm, echo times 2.3 ms and 4.6 ms, repetition time 76 ms, flip angle 70°, field of view 530 mm × 530 mm, acquisition matrix 216 × 177 and reconstruction matrix 480 × 480.

A previously reported, custom-made software used in-phase and opposed-phase information for automated image analysis [26]. SAT quantification relied on the accurate segmentation of outer and inner SAT boundaries on each individual slice. VAT areas were quantified by histogram analysis of the MRI signal distribution over an automatically computed VAT boundary (VAT “envelope”). The software highlighted all pixels with T1 signal intensities above a predefined histogram threshold (presumably fat). An experienced reader then reviewed the boundaries and threshold on each slice and made proper adjustments where necessary. The resulting adipose tissue areas and volumes (pixel areas multiplied with effective slice thickness) are then provided as a formatted output file. Patients with any apparent cropping of abdominal SAT regions at the (technically limited) edge of the field of view were excluded from further analysis.

Throughout the text, acronyms SAT and TAT strictly relate to the abdominopelvic (abdominal) compartment only (not the whole body).

### Anthropometric parameters

Anthropometric parameters included the measured circumferences of the waist (WC) and hip (HC), the waist-to-hip ratio (WHR), the normalized waist-to-height ratio (WHtR) and the (hypothetical) hip-to-height ratio (HHtR).

#### Numerical and statistical analysis

Statistical BMI distributions for each sex and combined were tested for normality using Shapiro–Wilk (S–W) and Kolmogorov–Smirnov (K–S) tests. Linear regressions between anthropometric parameters (*AP*, independent variable) and all adipose tissue volumes (*V*_SAT_, *V*_VAT_ and *V*_TAT_) were used to determine slope *m*_ap_, intercept *b*_ap_, Pearson’s correlation coefficient *r* and coefficient of determination *R*^2^. The equation to estimate one of the adipose tissue volumes *V*_XAT_ (with X = S,V or T) then becomes

1 $${{{\mathrm{V}}}}^\sim _{{{{\mathrm{XAT}}}}} = {\it{m}}_{{\it{ap}}}{\it{AP}} + {\it{b}}_{{\it{ap}}}$$

with anthropometric parameter *AP* (BMI, WC, HC, WHR, WHtR or HHtR) and parameters *m*_ap_ and *b*_ap_ according to the corresponding set of regression parameters for a given *AP* and compartment XAT–the tilde (^~^) denoting the estimated (computed) XAT volume. As an independent measure of agreement, the standard deviation *s*_d%_ of the percent differences (*V*^~^_XAT_ – *V*_XAT_) / *V*_XAT_ ∙ 100% between estimated and measured *V*_XAT_ is provided.

## Results

**Fig. 1:**
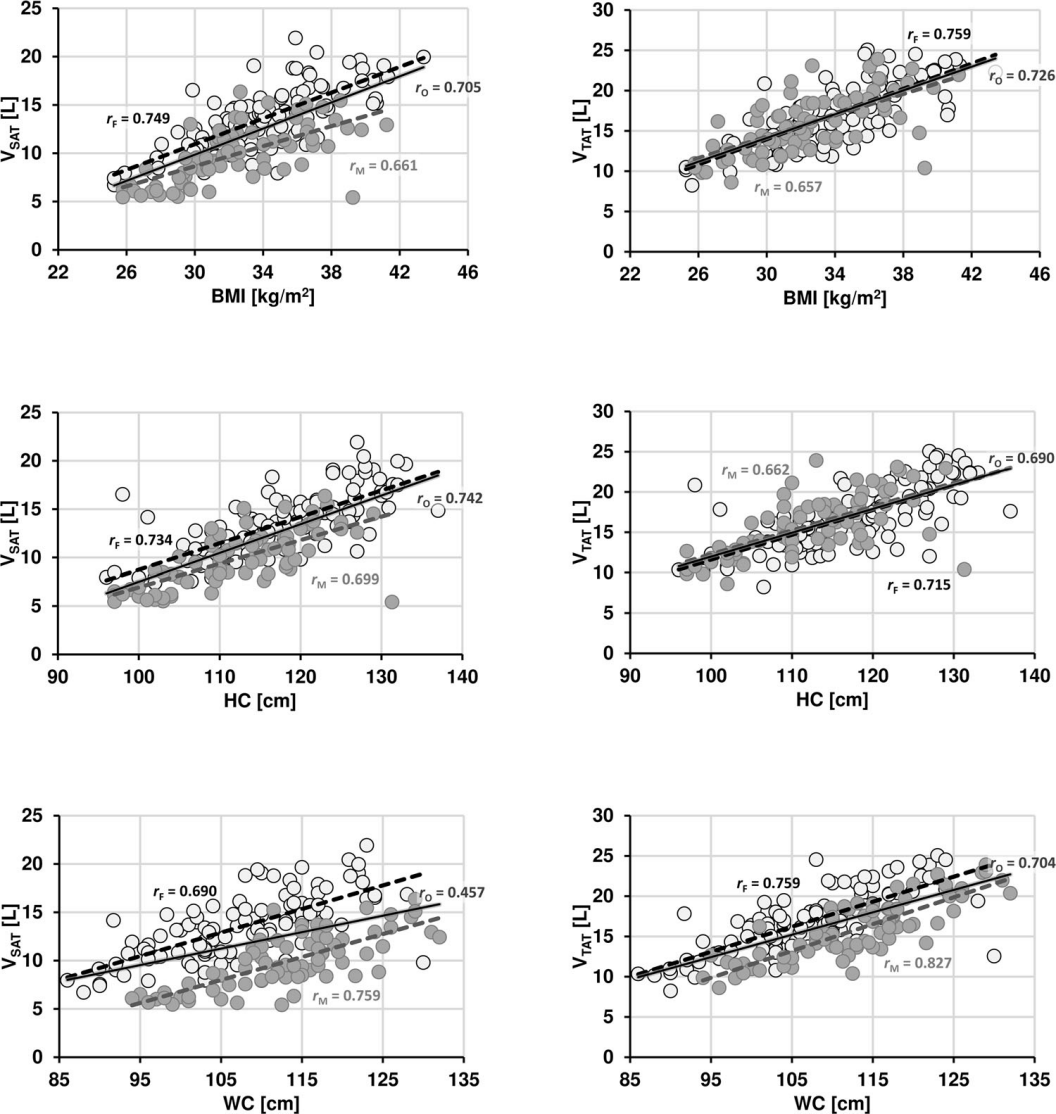
Regression plots of anthropometric parameters–BMI (top row), HC (middle row) and WC (bottom row)–and abdominal adipose tissue volumes SAT (left column) and TAT (right column). Data points and linear fits are shown for females (white circles, dashed black line), males (gray circles, dashed gray line) and both sexes (solid gray line) together with Pearson’s correlation coefficients *r*_F_, *r*_M_ and *r*_O_, for females, males and overall, respectively.

Table 1 provides an overview of the patient characteristics for each sex. Fig. 1 shows the regression data of selected anthropometric parameters (BMI, HC and WC) with SAT and TAT. The overlaid regression lines for females and males are typically offset against each other, most notably for SAT, suggesting sex-specific differences.

**Table 1:** Characteristics of the study population.

	women (108)	men (73)
Age [years]	50.9 ± 12.4 (24.2–86.7)	57.2 ± 11.6 (30.6–78.0)
Height [m]	1.64 ± 0.06 (1.51–1.82)	1.77 ± 0.06 (1.61–1.90)
Weight [kg]	91.1 ± 11.3 (63.0–118.7)	101.1 ± 12.6 (78.2–138.1)
BMI [kg/m^2^]	33.8 ± 3.7 (25.3–43.4)	32.3 ± 3.7 (25.8–41.2)
SAT [L]	13.47 ± 3.31 (6.71–21.94)	9.87 ± 2.88 (5.42–16.36)
VAT [L]	3.46 ± 1.51 (0.69–7.79)	5.96 ± 1.84 (1.71–11.19)
TAT [L]	16.93 ± 3.84 (8.24–25.06)	15.83 ± 3.70 (8.62–23.92)
WC [cm]	107.3 ± 9.4 (86.0–130.0)	112.9 ± 9.2 (94.0–132.0)
HC [cm]	117.3 ± 8.9 (96.0–137.0)	112.1 ± 8.3 (97.0–131.3)

Table 2 provides a summary of the sex-specific linear-regression parameters, Pearson correlation coefficients (ordinary and squared) as well as standard deviations of the percent differences for the estimation of abdominal SAT and TAT volumes. All anthropometric measures were inappropriate for predicting VAT volume with the highest sex-specific *R*^2^ values just 0.182 for females (WHtR) and 0.226 for males (WC) (see supplementary material for details). All following statements therefore apply to SAT and TAT estimation only. Parameter WHR had the weakest agreement of all six parameters (*R*^2^ < 0.100). The sex-specific parameters always outperformed the joint estimation meaning higher *R*^2^ and better (smaller) *s*_d%_. For females, the strongest associations and smallest deviations for estimating SAT volumes were observed for BMI and HC, while BMI was superior in predicting TAT. For males, SAT and TAT volumes could both be best estimated by WC. Normalization of circumferences (HC and WC) to height (HHtR and WHtR) did not improve agreement.

**Table 2:** Sex-specific measures of agreement and linear regression (fit) parameters (slope *m*_ap_ and intercept *b*_ap_) between anthropometric (ap) measures and abdominal adipose tissue volumes SAT and TAT (reference).

	Females (108)				Males (73)				Overall (181)			
	*r* (*R*^2^)	*s*_d%_ [%]	*m*_ap_	*b*_ap_ [L]	*r* (*R*^2^)	*s*_d%_ [%]	*m*_ap_	*b*_ap_ [L]	*r* (*R*^2^)	*s*_d%_ [%]	*m*_ap_	*b*_ap_ [L]
SAT												
BMI	**0.749 (0.560)**	17.5	0.666 Lm^2^/kg	−9.049	0.661 (0.436)	26.0	0.519 Lm^2^/kg	−6.905	0.705 (0.497)	26.5	0.676 Lm^2^/kg	−10.426
HC	0.734 (0.539)	**17.2**	27.305 L/m	−18.564	0.699 (0.489)	26.4	24.329 L/m	−17.406	**0.742 (*0.551*)**	**25.1**	29.685 L/m	−22.185
WC	0.690 (0.477)	20.3	24.408 L/m	−12.729	**0.759 (0.575)**	**20.7**	23.785 L/m	−16.985	0.457 (*0.209*)	—	—	—
WHR	0.068 (*0.005*)	—	—	—	0.182 (*0.033*)	—	—	—	0.215 (*0.046*)	—	—	—
HHtR	0.643 (0.413)	19.5	37.079 L	−13.058	0.542 (*0.294*)	—	—	—	0.717 (0.515)	25.2	38.214 L	−14.080
WHtR	0.631 (*0.398*)	—	—	—	0.638 (0.407)	25.0	34.100 L	−11.927	0.611 (*0.374*)	—	—	—
TAT												
BMI	**0.759 (0.576)**	**15.6**	0.783 Lm^2^/kg	−9.530	0.657 (0.432)	19.6	0.663 Lm^2^/kg	−5.609	**0.726 (0.527)**	**17.4**	0.736 Lm^2^/kg	−7.964
HC	0.715 (0.511)	17.5	30.787 L/m	−19.188	0.662 (0.439)	19.7	29.592 L/m	−17.349	0.690 (0.475)	18.6	29.117 L/m	−17.035
WC	0.759 (0.576)	16.1	31.070 L/m	−16.421	**0.827 (0.684)**	**13.9**	33.323 L/m	−21.795	0.704 (0.496)	17.7	27.723 L/m	−13.867
WHR	0.056 (*0.003*)	—	—	—	0.139 (*0.019*)	—	—	—	0.001 (*0.0*)	—	—	—
HHtR	0.103 (*0.011*)	—	—	—	0.507 (*0.257*)	—	—	—	0.048 (*0.002*)	—	—	—
WHtR	0.034 (*0.001*)	—	—	—	0.570 (*0.324*)	—	—	—	0.061 (*0.004*)	—	—	—

*R*^*2*^, coefficient of determination; *s*_d%_, standard deviation of the percent differences, fit parameters and *s*_d%_ values for anthropometric measures with *R*^2^ < *0.400* (indicated in *italic type*) have been omitted (–); bold values indicate combination of smallest *s*_d%_ and highest *R*^2^ values within groups.

The breakdown into age groups showed some slight variation of the best parameters. For both younger females and males (<39 years), WC best predicted SAT and TAT volumes with smallest *s*_d%_. This also held for middle-aged males (40–59 years), whereas for females, HC and BMI outperformed WC for SAT and TAT prediction, respectively. For older patients (>60 years), BMI became favorable for both SAT and TAT prediction, only WC was better for TAT prediction in males. Over all patients, anthropometric measures also performed poorly in predicting VAT volume within the three age groups.

## Discussion

Body mass index is still the most common measure of body composition mainly because it is readily available, simple and sufficiently accurate for basic purposes. A general drawback of all anthropometric parameters is their inability to differentiate between lean and fat mass as well as between SAT and VAT [12–20]. BMI, for example, has been found to be less accurate for elderly patients whose muscles are atrophic [19] and tends to overestimate body fat in subjects with a muscular build [27].

The present study aimed to evaluate different anthropometric measures for the prediction of whole-abdominal adipose tissue volumes *V*_XAT_ (subcutaneous *V*_SAT_, visceral *V*_VAT_ and total *V*_TAT_) in patients with overweight and obesity. Our results show that sex-specific predictions were more accurate than overall ones. The strongest associations and smallest deviations in females were BMI and HC for SAT and BMI for TAT, whereas WC was the best parameter for both SAT and TAT in males. Normalization of these variables to the patients’ height did not improve the results. For the prediction of VAT volume, no anthropometric measure was found to be suitable.

Previous studies have stressed that BMI values can be easily recorded during clinical routine but might be useless for the assessment of abdominal fat compartments [12, 13, 16–20]. In one study of patients with coronary heart disease, Coutinho and coworkers have paradoxically observed an inverse association of BMI and mortality. The absence of more specific parameters than BMI might prevent patients with an increased metabolic risk profile but normal BMI to change their lifestyle [28].

Here, the value of BMI appears to be age-dependent. In older patients (60 years and above), BMI showed better results than circumferential measures for SAT prediction, whereas circumferences (HC for females and WC for males) were superior in our middle-aged patients (40–59 years). For the prediction of TAT volumes, best parameters depended on sex rather than age (BMI for females and WC for males) for both middle-age and old groups (>40 years). In the young group (<39 years), WC showed the smallest deviations independent of compartment (SAT and TAT) and sex.

Table 3 provides a summary of literature recommendations for SAT and TAT quantification. For SAT volume estimation, the smallest percent deviation *s*_d%_ for all patients was seen for HC (about 25%) closely followed by HHtR. Substantially better estimates may be obtained by a sex-specific consideration with minimum deviations of 17.2% to 17.5% for females (for HC and BMI) and 20.7% for males (for WC). BMI turned out to be slightly worse for sexes combined (vs. HC) and worse for males alone (vs. WC). In other words, BMI is suitable for female subjects, HC for female and mixed populations, whereas WC is best for male subjects.

**Table 3:** Summary of literature recommendations for SAT and TAT quantification.

Study	Population	Modality	Anthropometry	Mean BMI	Recommendation
SAT					
Ludescher et al. 2009 [25]	48 female 20 male	MRI	Skin fold BI WHR BMI SAT thickness D: M. rectus abd. to aorta	24	Skin fold (*r*_*p*_ = 0.662) BI (*r*_*p*_ = 0.7) Anthropometric data (*r*_*p*_ = 0.7-0.4)
Yim et al. 2010 [21]	(i) BMI ≥ 25 328 female 1498 male (ii) BMI < 25 1513 female 1761 male	CT	SAD TAD BMI WC	24	(i) F: SAD (*r* = 0.537), TAD (*r* = 0.769), BMI (*r* = 0.521), WC (*r* = 0.697) M: SAD (*r* = 0.527), TAD (*r* = 0.624), BMI (*r* = 0.402), WC (*r* = 0.689)
Mantatzis et al. 2014 [17]	(i) diabetic 38 male (ii) non-diabetic 38 male	MRI	WC BMI HC WHR BI	32 30	(i) M: WC (*r*_*p*_ = 0.708), BMI (*r*_*p*_ = 0.692), HC (*r*_*p*_ = 0.856), WHR (*r*_*p*_ = 0.138), BI (*r*_*p*_ = 0.565) (ii) M: WC (*r*_*p*_ = 0.785), BMI (*r*_*p*_ = 0.733), HC (*r*_*p*_ = 0.850), WHR (*r*_*p*_ = 0.333), BI (*r*_*p*_ = 0.644)
Incio et al., 2018 [29]	99 female (breast cancer)	CT	BMI	28	F: BMI (*r*_*s*_ = 0.799)
Present study, 2022	110 female 75 male	MRI	BMI HC WC	34 32	F:BMI (*r*_*p*_ = 0.768), HC (*r*_*p*_ = 0.752), WC (*r*_*p*_ = 0.708) M:BMI (*r*_*p*_ = 0.682), HC (*r*_*p*_ = 0.718), WC (*r*_*p*_ = 0.765)
TAT					
Eloi et al., 2017 [30]	(i) obese adolescents 13 female 11 male (ii) healthy adolescents 16 female 17 male	MRI	BMI WC WHR	26	Note: only TAT area at L3-L4 (i) BMI (*r* = 0.875), WC (*r* = 0.907), WHR (*r* = 0.862) (ii) BMI (*r* = 0.451), WC (*r* = 0.474), WHR (*r* = 0.133)
Browning et al., 2011 [22]	60 female 60 male	MRI	BMI WC (midpoint, umbilical)	27 28	F: BMI (*r*_*p*_ = 0.91), WC midpoint (*r*_*p*_ = 0.91), WC umbilical (*r*_*p*_ = 0.88) M: BMI (*r*_*p*_ = 0.92), WC midpoint (*r*_*p*_ = 0.96), WC umbilical (*r*_*p*_ = 0.96)
Neamat-Allah et al., 2015 [23]	594 female 598 male	MRI	BMI HC WC	26 27	F: BMI (*r*_*p*_ = 0.95), HC (*r*_*p*_ = 0.94), WC (*r*_*p*_ = 0.88) M: BMI (*r*_*p*_ = 0.89), HC (*r*_*p*_ = 0.84), WC (*r*_*p*_ = 0.91)
Al-Gindan et al., 2017 [24]	110 female 94 male	MRI	BMI HC WC WHR	25	F: BMI (*R*^2^ = 0.824), HC (*R*^2^ = 0.811), WC (*R*^2^ = 0.775), WHR (*R*^2^ = 0.025) M: BMI (*R*^2^ = 0.658), HC (*R*^2^ = 0.722), WC (*R*^2^ = 0.768), WHR (*R*^2^ = 0.355)
Present study, 2022	110 female 75 male	MRI	BMI HC WC	34 32	F: BMI (*r*_*p*_ = 0.771), HC (*r*_*p*_ = 0.730), WC (*r*_*p*_ = 0.769) M: BMI (*r*_*p*_ = 0.681), HC (*r*_*p*_ = 0.688), WC (*r*_*p*_ = 0.839)

BI bioelectrical impedance analysis, D diameter, F female, M male, SAD sagittal abdominal diameter, TAD transverse abdominal diameter.

Contrary to our results, Mantatzis et al. have seen the highest correlation of SAT volume with HC followed by WC [17] but their population was smaller and consisted of males only. In a female population, Incio et al. have found BMI to be a good predictor for SAT, in line with our results, but their population consisted of cancer patients with a lower mean BMI (28 vs. 34 kg/m^2^) and they reported the Spearman coefficient (unlike all other works) [29]. In a subgroup of subjects with BMI ≥ 25 kg/m^2^, Yim et al. have also identified WC as best parameter for TAT estimation in males whereas BMI correlated only moderately in females. This might be due to the different population (Asian vs. European) or different BMI distribution – although the mean BMI is not reported for that subgroup [21].

For TAT volume estimation, best agreement for both sexes was seen for BMI (17.4%) closely followed by WC. Again, a sex-specific consideration is recommended reducing the deviations to around 16% for females (for BMI and WC) and under 14% for males (for WC). Eloi and coworkers have also found WC to be a good predictor of TAT for patients with obesity but not for healthy adolescents with normal BMI. In addition, both groups were small and comprised of both sexes [30]. Similar to our work, three sex-dependent analyses have reported BMI (females) and WC (males) as best estimators for TAT [22–24] but the study cohorts were rather different. Neamat-Allah and coworkers, for example, have included cancer patients (from a large study network, EPIC-Germany), which should introduce a bias in fat distribution and BMI range [23]. In the analysis of Browning et al. [22], only 80 (out of 120) patients had a BMI of at least 25 kg/m^2^. In line with our results, Al-Gindan et al. have identified BMI and WC as best parameters for TAT estimation in females and males [24]. Their *R*^2^ values were slightly higher than the ones here but were derived in a population with a substantially lower mean BMI (25 kg/m^2^), which seems to matter in the light of the above findings from Eloi et al. [30].

VAT is an important indicator of adverse cardiometabolic health. Our results show that all anthropometric variables performed poorly in predicting VAT volume. A previous study by Linder et al. showed that VAT volumes in patients with morbid obesity can be predicted rather reliably by simply multiplying the segmented VAT area at a gender-specific lumbar reference level with a fixed scaling factor and effective slice thickness [31].

Sex differences in body shape are often put forth to imply a visceral type of adiposity in men and a subcutaneous one in women–predominantly in the gluteofemoral region [32]. This might partially explain why SAT volume correlates strongly with HC in women and with WC in men. Circumferences can be quantified easily but need to be standardized in terms of measurement level (between lower costal margin and iliac crest) and subject position (lying or standing), especially for high-grade obesity [18, 33]. Although the waist circumference cannot discriminate between VAT and SAT, there are reports in the literature where WC correlated better with either SAT [12, 17, 21] or VAT [13, 18, 34–37]. Despite this inconsistency, all studies seem to agree on the priority of WC over BMI.

Normalizing variables to participants’ height did not improve the predictions. The ratio of waist circumference to height (WHtR) has been suggested in the mid-1990 s already [38–40] to improve estimations of the metabolic risk in relatively small or relatively large patients [41]. Follow-up studies by Danish and Japanese groups, however, did not support such an approach [42, 43]. The ratio of waist circumference to hip circumference (WHR) has been considered as well but might be limited by its design. A weight gain, for example, might lead to an increase in both circumferences and effectively leave the ratio unchanged [27]. For two individuals with the same WC, the WHR will be higher for the one with the smaller HC [20]. The WHR therefore appears to be inappropriate for the assessment of abdominal obesity and metabolic risks.

There is considerable interest in identifying robust predictors for risk stratification and clinical follow-up of obesity-associated metabolic effects. Potential applications of our method include longitudinal obesity studies in which fat content and distribution must be controlled or studies evaluating conservative or surgical procedures in which body fat is used as a predictive biomarker. With limited health resources, it is a challenge to distinguish early between patients with a healthy metabolic phenotype and those with an elevated cardio-metabolic risk profile.

Blüher has described the metabolically healthy phenotype (MHO) as a subentity of obesity in which excessive body fat accumulation does not lead to adverse metabolic effects [44]. Such individuals are characterized by higher subcutaneous fat mass and lower visceral and ectopic fat storage. However, a targeted quantification and follow-up of individual abdominal fat compartments is important to distinguish these metabolic phenotypes. In future guidelines, such an approach could be of great use for cardiometabolic risk stratification of patients with overweight or obesity. We believe that the present findings have a high practical value and may also encourage more clinical work in obesity.

This study is limited by its retrospective and single-center design; a generalization of the findings is therefore difficult. The results for male subjects should be interpreted with some care given their smaller fraction within the overall study group. While interactive segmentation is a standard method of tissue volumetry, variation between different observers should be taken into consideration. A histopathological reference was not part of the protocol. Datasets were taken from prior studies and might not be representative for a general population of patients with obesity. This also applies to the factor ethnicity. The BMI range of our study patients was relatively large. A common limitation of studies involving volumetric segmentation of tissue compartments so far is the limited sample size, which leaves little room for detailed subgroup analyses. It should therefore be noted that the values and findings here may not be directly transferable to other study cohorts.

In conclusion, we recommend the use of sex-specific parameters–BMI and HC for females and WC for males–for the estimation of abdominal SAT and TAT volumes in patients with overweight and obesity. Pooled measures (females and males together) were less reliable–a moderate agreement was seen for HC (SAT) and BMI (TAT).

## Supplementary information

Supplemental Table 1

Supplemental Table 2

Supplemental Figure 1

Supplemental Figure 2

Supplementary Figure Legends

## Data Availability

The datasets generated during and/or analysed during the current study are available from the corresponding author on reasonable request.
