# Supplementary material for: Anthropometric estimators of abdominal fat volume in adults with overweight and obesity
Source: Int J Obes (Lond). 2023 Feb 7;47(4):306–12. doi: 10.1038/s41366-023-01264-x (PMC10113142; doi:10.1038/s41366-023-01264-x)
Supplement: Supplementary file 1 — Supplemental Table 1 [file 41366_2023_1264_MOESM1_ESM.docx]

Supplemental Table 1: Sex-specific measures of agreement and linear regression (fit) parameters (slope *m*_ap_ and intercept *b*_ap_) between anthropometric (ap) measures and abdominal adipose tissue volumes VAT (reference)

|  | **Females (108)** | | | | **Males (73)** | | | | **Overall (181)** | | | |
| --- | --- | --- | --- | --- | --- | --- | --- | --- | --- | --- | --- | --- |
|  | ***r* (*R*^2^)** | ***s*_d%_ [%]** | ***m*_ap_** | ***b*_ap_ [L]** | ***r* (*R*^2^)** | ***s*_d%_ [%]** | ***m*_ap_** | ***b*_ap_ [L]** | ***r* (*R*^2^)** | ***s*_d%_ [%]** | ***m*_ap_** | ***b*_ap_ [L]** |
| **VAT** |  |  |  |  |  |  |  |  |  |  |  |  |
| BMI | 0.287 (0.083) | 63.8 | 0.116 Lm^2^/kg | -0.477 | 0.287 (0.082) | 47.6 | 0.144 Lm^2^/kg | 1.296 | 0.111 (0.012) | 81.3 | 0.060 Lm^2^/kg | 2.462 |
| HC | 0.206 (0.042) | 68.0 | 3.481 L/m | -0.623 | 0.237 (0.056) | 48.2 | 5.263 L/m | 0.058 | 0.003 (0.0) | 83.8 | -0.077 L/m | 4.557 |
| WC | 0.414 (0.171) | 56.6 | 6.663 L/m | -3.691 | 0.476 (0.226) | 45.6 | 9.537 L/m | -4.810 | 0.507 (0.257) | 62.8 | 10.766 L/m | -7.330 |
| \| WHR \| \| --- \| \| HHtR \| \| WHtR \| | 0.291 (*0.085*) | 62.9 | 6.641 L | -2.625 | 0.372 (*0.139*) | 43.2 | 12.365 L | -6.506 | 0.559 (*0.312*) | 59.4 | 14.997 L | -9.829 |
| \| HHtR \| \| --- \| \| HHtR \| \| WHtR \| | 0.215 (0.046) | 67.5 | 5.650 L | -0.582 | 0.120 (*0.014*) | 47.4 | 4.322 L | 3.214 | 0.239 (*0.057*) | 83.7 | -7.246 L | 9.417 |
| \| WHtR \| \| --- \| \| HHtR \| \| WHtR \| | 0.426 (0.182) | 55.9 | 11.118 L | -3.816 | 0.366 (0.134) | 45.8 | 12.518 L | -2.043 | 0.236 (0.056) | 75.1 | 8.548 L | -1.074 |
| *R^2^*, coefficient of determination; *s*_d%_, standard deviation of the percent differences | | | | | | | | | | | | |
