# Supplementary material for: Anthropometric estimators of abdominal fat volume in adults with overweight and obesity
Source: Int J Obes (Lond). 2023 Feb 7;47(4):306–12. doi: 10.1038/s41366-023-01264-x (PMC10113142; doi:10.1038/s41366-023-01264-x)
Supplement: Supplementary file 2 — Supplemental Table 2 [file 41366_2023_1264_MOESM2_ESM.docx]

Supplemental Table 2: Age-and sex-specific measures of agreement and linear regression (fit) parameters (slope *m*_ap_ and intercept *b*_ap_) between anthropometric (ap) measures and abdominal adipose tissue volumes SAT, TAT and VAT (reference)

**Age <39 years**

|  | **Females (19)** | | | | **Males (6)** | | | |
| --- | --- | --- | --- | --- | --- | --- | --- | --- |
|  | ***r* (*R*^2^)** | ***s*_d%_ [%]** | ***m*_ap_** | ***b*_ap_ [L]** | ***r* (*R*^2^)** | ***s*_d%_ [%]** | ***m*_ap_** | ***b*_ap_ [L]** |
| **SAT** |  |  |  |  |  |  |  |  |
| BMI | 0.665 (0.442) | 16.8 | 0.577 Lm^2^/kg | -5.441 | 0.257 (0.066) | 13.2 | 0.137 Lm^2^/kg | 7.660 |
| HC | 0.673 (0.454) | 16.7 | 27.913 L/m | -18.012 | 0.014 (0.0) | 15.8 | -0.432 L/m | 12.724 |
| WC | **0.799 (0.639)** | **12.0** | 24.296 L/m | -10.895 | **0.856 (0.733)** | **6.3** | 35.098 L/m | -27.826 |
| WHR | 0.569 (*0.323*) | 20.8 | 33.400 L | -15.005 | 0.609 (*0.370*) | 8.6 | 20.013 L | -6.987 |
| HHtR | 0.505 (0.255) | 19.1 | 33.031 L | -8.699 | 0.103 (*0.011*) | 13.7 | 4.139 L | 9.482 |
| WHtR | 0.736 (0.541) | 13.8 | 38.940 L | -10.185 | 0.690 (0.476) | 7.4 | 33.443 L | -8.902 |
| **TAT** |  |  |  |  |  |  |  |  |
| BMI | 0.686 (0.470) | 16.6 | 0.675 Lm^2^/kg | -6.657 | 0.375 (0.140) | 21.2 | 0.285 Lm^2^/kg | 26.336 |
| HC | 0.609 (0.371) | 18.7 | 28.596 L/m | -16.566 | **0.561 (0.315)** | 21.0 | -24.445 L/m | 45.948 |
| WC | **0.787 (0.619)** | **13.3** | 27.110 L/m | -11.648 | 0.276 (0.076) | **13.2** | 16.170 L/m | -1.602 |
| \| WHR \| \| --- \| \| HHtR \| \| WHtR \| | 0.309 (*0.096*) | 22.4 | -22.693 L | 38.163 | 0.465 (*0.216*) | 27.8 | 42.430 L | -25.035 |
| \| HHtR \| \| --- \| \| HHtR \| \| WHtR \| | 0.033 (0.001) | 22.0 | -2.268 L | 19.296 | 0.221 (*0.049*) | 25.8 | 32.912 L | -3.400 |
| WHtR | 0.175 (0.031) | 21.9 | -9.486 L | 23.891 | 0.681 (0.464) | 22.0 | 102.155 L | -46.672 |
| **VAT** |  |  |  |  |  |  |  |  |
| BMI | 0.459 (0.211) | - | - | - | **0.782 (0.612)** | - | - | - |
| HC | 0.067 (0.005) | - | - | - | 0.778 (0.605) | - | - | - |
| WC | 0.378 (0.143) | **-** | - | - | 0.456 (0.208) | - | - | - |
| \| WHR \| \| --- \| \| HHtR \| \| WHtR \| | **0.533 (*0.284*)** | - | - | - | 0.353 (*0.125*) | **-** | - | - |
| \| HHtR \| \| --- \| \| HHtR \| \| WHtR \| | 0.086 (0.007) | - | - | - | 0.879 (*0.773*) | - | - | - |
| \| WHtR \| \| --- \| \| HHtR \| \| WHtR \| | 0.415 (0.172) | - | - | - | 0.645 (0.416) | - | - | - |

**Age 40 - 59 years**

|  | **Females (61)** | | | | **Males (34)** | | | |
| --- | --- | --- | --- | --- | --- | --- | --- | --- |
|  | ***r* (*R*^2^)** | ***s*_d%_ [%]** | ***m*_ap_** | ***b*_ap_ [L]** | ***r* (*R*^2^)** | ***s*_d%_ [%]** | ***m*_ap_** | ***b*_ap_ [L]** |
| **SAT** |  |  |  |  |  |  |  |  |
| BMI | 0.658 (0.443) | 20.1 | 0.697 Lm^2^/kg | -10.072 | 0.392 (0.153) | - | - | - |
| HC | **0.679 (0.461)** | **18.2** | 25.445 L/m | -16.609 | 0.428 (0.183) | - | - | - |
| WC | 0.643 (0.414) | 21.8 | 22.988 L/m | -11.394 | **0.649 (0.421)** | **22.9** | 21.012 L/m | -13.401 |
| WHR | 0.065 (*0.004*) | 26.9 | 2.852 L | 10.883 | 0.337 (*0.114*) | 28.1 | 15.360 L | -4.421 |
| HHtR | 0.584 (0.341) | 19.9 | 33.787 L | -10.814 | 0.249 (*0.062*) | - | - | - |
| WHtR | 0.567 (0.322) | 22.7 | 32.003 L | -7.575 | 0.529 (0.280) | 26.8 | 29.676 L | -8.393 |
| **TAT** |  |  |  |  |  |  |  |  |
| BMI | **0.731 (0.534)** | **17.0** | 0.945 Lm^2^/kg | -14.953 | 0.515 (0.265) | 22.9 | 0.576 Lm^2^/kg | -2.222 |
| HC | 0.666 (0.443) | 19.3 | 30.488 L/m | -19.045 | 0.455 (0.207) | 24.3 | 23.837 L/m | -10.335 |
| WC | 0.721 (0.519) | 18.1 | 31.457 L/m | -17.032 | **0.756 (0.572)** | **16.9** | 33.208 L/m | -21.295 |
| \| WHR \| \| --- \| \| HHtR \| \| WHtR \| | 0.046 (*0.002*) | 26.6 | 2.576 L | 14.551 | 0.041 (*0.002*) | 22.2 | 2.866 L | 12.990 |
| \| HHtR \| \| --- \| \| HHtR \| \| WHtR \| | 0.070 (0.005) | 26.7 | -4.456 L | 20.116 | 0.487 (*0.237*) | 17.7 | 32.848 L | -4.988 |
| \| WHtR \| \| --- \| \| HHtR \| \| WHtR \| | 0.006 (0.0) | 26.7 | -0.431 L | 17.207 | 0.514 (0.265) | 18.2 | 34.945 L | -6.577 |
| **VAT** |  |  |  |  |  |  |  |  |
| BMI | 0.487 (0.237) | - | - | - | 0.427 (0.182) | **-** | - | - |
| HC | 0.279 (0.078) | - | - | - | 0.262 (0.069) | - | - | - |
| WC | 0.491 (0.241) | - | - | - | **0.524 (0.275)** | - | - | - |
| \| WHR \| \| --- \| \| HHtR \| \| WHtR \| | 0.268 (*0.072*) | - | - | - | 0.358 (*0.129*) | **-** | - | - |
| \| HHtR \| \| --- \| \| HHtR \| \| WHtR \| | 0.291 (0.085) | - | - | - | 0.113 (*0.013*) | - | - | - |
| \| WHtR \| \| --- \| \| HHtR \| \| WHtR \| | **0.494 (0.244)** | **-** | - | - | 0.412 (0.170) | - | - | - |

**Age >60 years**

|  | **Females (28)** | | | | **Males (33)** | | | |
| --- | --- | --- | --- | --- | --- | --- | --- | --- |
|  | ***r* (*R*^2^)** | ***s*_d%_ [%]** | ***m*_ap_** | ***b*_ap_ [L]** | ***r* (*R*^2^)** | ***s*_d%_ [%]** | ***m*_ap_** | ***b*_ap_ [L]** |
| **SAT** |  |  |  |  |  |  |  |  |
| BMI | **0.908 (0.825)** | **10.9** | 0.633 Lm^2^/kg | -8.307 | **0.865 (0.748)** | **16.2** | 0.584 Lm^2^/kg | -9.581 |
| HC | 0.878 (0.771) | 13.8 | 28.396 L/m | -20.139 | 0.831 (0.691) | 16.9 | 27.837 L/m | -21.520 |
| WC | 0.774 (0.599) | 17.7 | 26.925 L/m | -16.114 | 0.853 (0.728) | 16.8 | 20.870 L/m | -14.546 |
| WHR | 0.115 (*0.013*) | - | - | - | 0.456 (*0.208*) | - | - | - |
| HHtR | 0.847 (0.718) | 16.9 | 40.838 L | -16.410 | 0.711 (*0.505*) | 22.3 | 34.162 L | -12.654 |
| WHtR | 0.789 (0.622) | 19.1 | 43.201 L | -15.746 | 0.802 (0.643) | 18.8 | 31.206 L | -11.253 |
| **TAT** |  |  |  |  |  |  |  |  |
| BMI | **0.916 (0.839)** | **10.2** | 0.766 Lm^2^/kg | -8.519 | 0.763 (0.583) | 15.5 | 0.710 Lm^2^/kg | -7.564 |
| HC | 0.881 (0.776) | 11.6 | 34.187 L/m | -22.630 | 0.847 (0.717) | 13.9 | 39.058 L/m | -27.653 |
| WC | 0.837 (0.700) | 13.0 | 34.960 L/m | -20.470 | **0.893 (0.797)** | **10.1** | 30.088 L/m | -18.749 |
| \| WHR \| \| --- \| \| HHtR \| \| WHtR \| | 0.285 (*0.081*) | 29.3 | 17.011 L | 0.872 | 0.170 (*0.029*) | 27.9 | 10.396 L | 4.784 |
| \| HHtR \| \| --- \| \| HHtR \| \| WHtR \| | 0.304 (0.092) | 29.4 | -20.852 L | 30.924 | 0.576 (*0.332*) | 22.9 | 38.268 L | -8.968 |
| \| WHtR \| \| --- \| \| HHtR \| \| WHtR \| | 0.041 (0.002) | - | - | - | 0.638 (0.407) | 21.2 | 38.755 L | -9.299 |
| **VAT** |  |  |  |  |  |  |  |  |
| BMI | 0.447 (0.200) | - | - | - | 0.263 (0.069) | - | - | - |
| HC | 0.419 (0.176) | - | - | - | 0.471 (0.222) | 29.7 | 11.221 L/m | -6.133 |
| WC | **0.540 (0.292)** | **29.2** | 8.035 L/m | -4.356 | **0.529 (0.280)** | **25.1** | 9.218 L/m | -4.204 |
| \| WHR \| \| --- \| \| HHtR \| \| WHtR \| | 0.164 (*0.027*) | - | - | - | 0.355 (*0.126*) | - | - | - |
| \| HHtR \| \| --- \| \| HHtR \| \| WHtR \| | 0.320 (0.102) | - | - | - | 0.262 (*0.069*) | - | - | - |
| \| WHtR \| \| --- \| \| HHtR \| \| WHtR \| | 0.458 (0.210) | - | - | - | 0.378 (0.143) | 28.0 | 10.468 L | -0.674 |
| *R^2^*, coefficient of determination; *s*_d%_, standard deviation of the percent differences, fit parameters and *s*_d%_ values for anthropometric measures with *s*_d%_ < ***30.0*** have been omitted (–); bold values indicate combination of smallest *s*_d%_ and highest *R*^2^ values within groups | | | | | | | | |
