# Supplementary figures and images for: Anthropometric estimators of abdominal fat volume in adults with overweight and obesity
Source: Int J Obes (Lond). 2023 Feb 7;47(4):306–12. doi: 10.1038/s41366-023-01264-x (PMC10113142; doi:10.1038/s41366-023-01264-x)

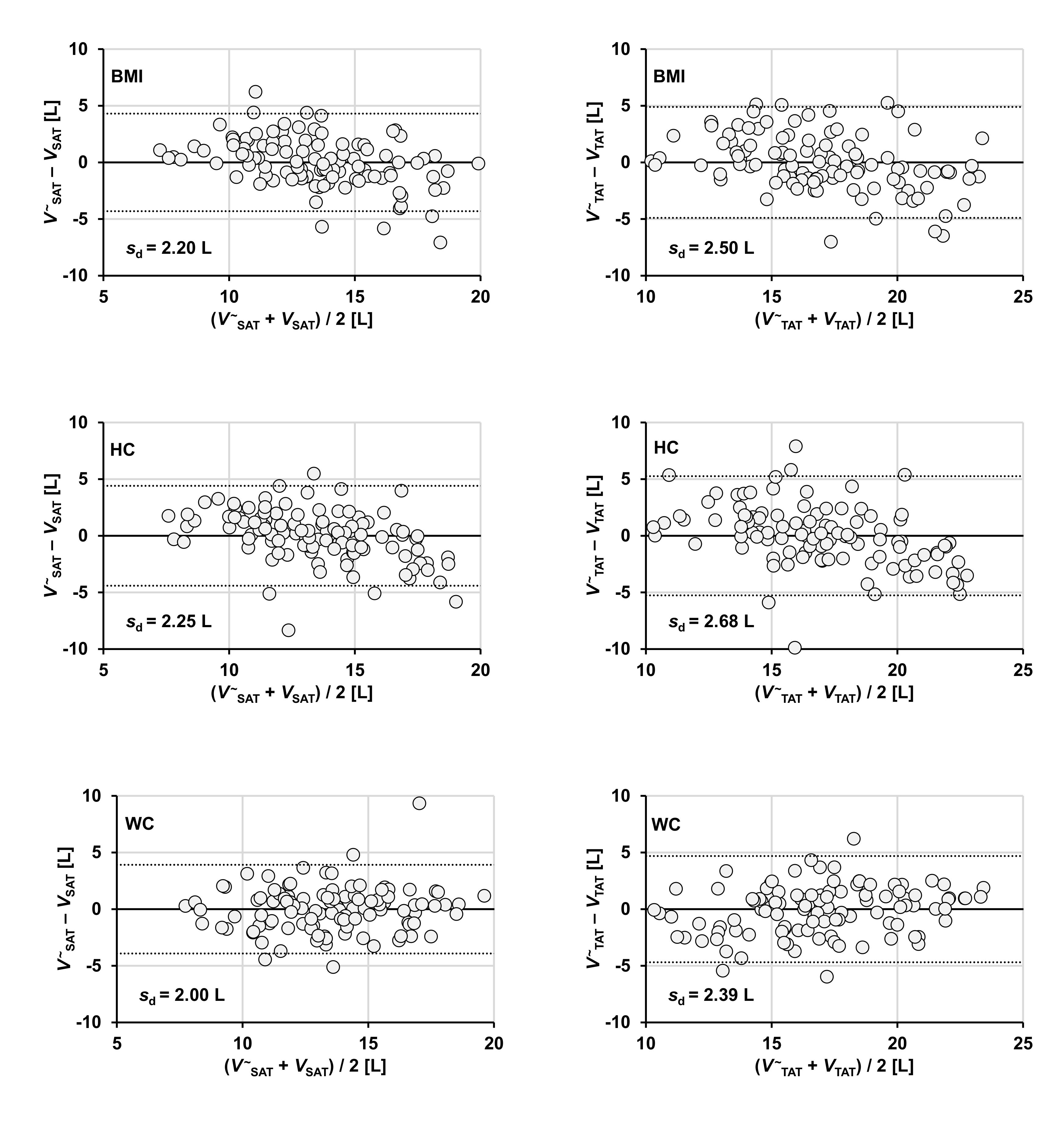

Supplement: Supplementary file 3 — Supplemental Figure 1 [file 41366_2023_1264_MOESM3_ESM.png]

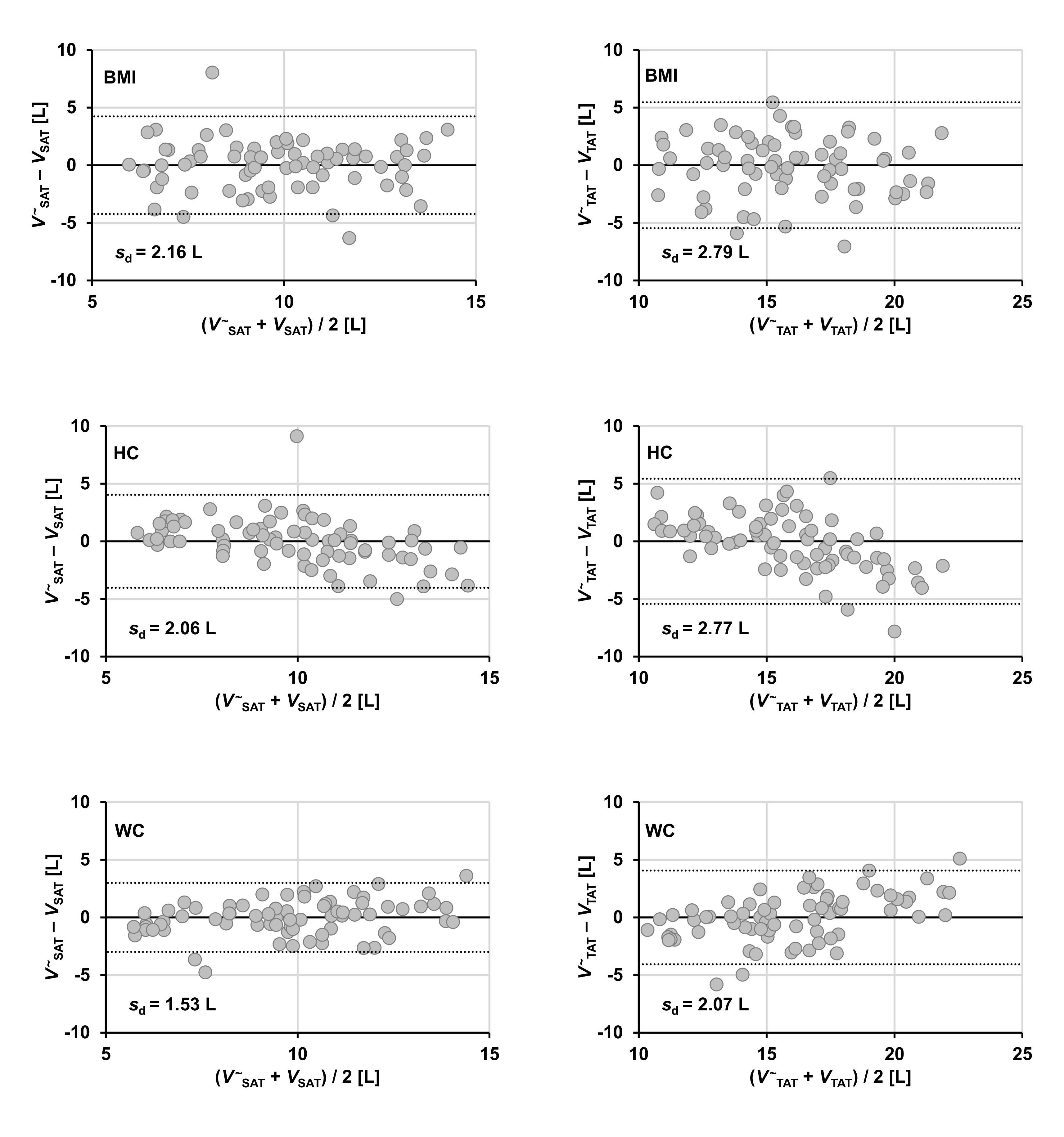

Supplement: Supplementary file 4 — Supplemental Figure 2 [file 41366_2023_1264_MOESM4_ESM.png]
