# Supplementary material for: Anthropometric estimators of abdominal fat volume in adults with overweight and obesity
Source: Int J Obes (Lond). 2023 Feb 7;47(4):306–12. doi: 10.1038/s41366-023-01264-x (PMC10113142; doi:10.1038/s41366-023-01264-x)
Supplement: Supplementary file 5 — Supplementary Figure Legends [file 41366_2023_1264_MOESM5_ESM.docx]

**Legend for Supplemental Figures**

Bland-Altman analyses of abdominal subcutaneous (SAT, left column) and total (TAT, right column) adipose tissue volumes between estimations (*V~*) from anthropometric parameters (BMI, HC, WC; top to bottom row) and measured volumes (*V*) for female (**Suppl. Fig. 1**) and male subjects (**Suppl. Fig. 2**). Dotted lines indicate 95% limits of agreement and *s*_d_ is the standard deviation of the differences between estimated and measured volumes. See text for further details.
